# Influence of Rhythmic Grouping on Duration Perception: A Novel Auditory Illusion

**DOI:** 10.1371/journal.pone.0054273

**Published:** 2013-01-18

**Authors:** Eveline Geiser, John D. E. Gabrieli

**Affiliations:** Department of Brain and Cognitive Sciences and McGovern Institute for Brain Research, Massachusetts Institute of Technology, Cambridge, Massachusetts, United States of America; Duke University, United States of America

## Abstract

This study investigated a potential auditory illusion in duration perception induced by rhythmic temporal contexts. Listeners with or without musical training performed a duration discrimination task for a silent period in a rhythmic auditory sequence. The critical temporal interval was presented either *within* a perceptual group or *between* two perceptual groups. We report the just-noticeable difference (difference limen, DL) for temporal intervals and the point of subjective equality (PSE) derived from individual psychometric functions based on performance of a two-alternative forced choice task. In musically untrained individuals, equal temporal intervals were perceived as significantly longer when presented *between* perceptual groups than *within* a perceptual group (109.25% versus 102.5% of the standard duration). Only the perceived duration of the *between*-group interval was significantly longer than its objective duration. Musically trained individuals did not show this effect. However, in both musically trained and untrained individuals, the relative difference limens for discriminating the comparison interval from the standard interval were larger in the *between*-groups condition than in the *within*-group condition (7.3% vs. 5.6% of the standard duration). Thus, rhythmic grouping affected sensitivity to duration changes in all listeners, with duration differences being harder to detect at boundaries of rhythm groups than *within* rhythm groups. Our results show for the first time that temporal Gestalt induces auditory duration illusions in typical listeners, but that musical experts are not susceptible to this effect of rhythmic grouping.

## Introduction

To efficiently perceive and interact with our environment, we tend to order sensory input in regular, recurring, and simple units. This most fundamental principle of Gestalt perception posits that global perceptual organization is achieved on the basis of the similarity and the spatial and temporal proximity of sensory units [1]. In auditory experience, such as the perception of noise, speech, or music, acoustic signals usually unfold in time, and thus comprise a temporal structure [2]–[5]. The temporal structure in music or speech is sometimes referred to as rhythm. It spontaneously results in perceptual grouping, which in turn facilitates efficient processing, e.g., of speech [6]. Here, we investigated a potential duration illusion induced by rhythmic grouping. Furthermore, we investigated whether musical training affects the influence of rhythm processing on duration perception.

In a rhythmical sequence with un-equal inter-tone intervals, most people perceive tones that are closer together as one group. This reflects “proximity grouping” – the larger the difference in proximity between tones the more likely close tones will be perceived as a group. We assumed that the rhythmic grouping of tones induces perceptual mechanisms that highlight group boundaries, and hypothesized that the *rhythm-induced perceptual Gestalt* might affect the subjective perception of inter-tone intervals. The idea that perceptual grouping could influence duration perception was already suggested in 1903 in the context of “subjective rhythmization” [7], [8]. This occurs when listeners are presented with an isochronous sequence of identical sounds in which they reported hearing alternating accentuation resulting in groups of two tones. Some individuals also reported hearing alternating long and short temporal intervals between the sounds. This is an illusion, because the intervals are objectively identical. An everyday example of this phenomenon is the “tick-tock” one hears when listening to a clock. Inferior duration estimation between perceptual groups, marked by pitch differences, has been attributed to an illusory elongation of a silent interval between groups [9], [10]. However, to our knowledge this potential subjective elongation of a temporal interval between perceptual groups has never been tested directly.

Distortions of the subjective experience of time and the perception of event durations have been reported in both the auditory and visual domains [11]. For example, the temporal dynamics, structure, and magnitude of a visual stimulus can affect its perceived duration [12], [13]. In the auditory domain, distortions have been reported for three-tone sequences in which the inter-tone interval between the second and third tones is longer than the interval between the first and second tones - the duration of the longer time interval is underestimated. This phenomenon is commonly referred to as time-shrinking [14], [15] and has a parallel that is referred to as time-stretching, which can be induced through the presentation of filled, instead of empty, time intervals [16]. Furthermore, reports addressing the chronotopic categorical clustering of rhythm perceptions have indicated that the temporal Gestalt could influence local perceptions of duration [17]–[19]. We asked whether such a “time warping” would occur in the context of rhythm-induced perceptual grouping by investigated the influence of rhythm on duration perception.

Musical expertise has been shown to influence temporal processing of rhythmic tone sequences, which raises the possibility that musicians compared to musically naïve participants would display different effects of rhythm on duration perception. Specifically, musicians compared to non-musicians show more efficient and refined processing of auditory temporal patterns as evidenced by the processing of temporally expected tone omissions [20], [21] and musical beat perception [22], [23]. Based on these findings we assumed that musicians might be more sensitive to rhythmic grouping structure in a tone sequence. Two alternative hypotheses about the influence of increased sensitivity to rhythm related to musical expertise were possible. Increased sensitivity to rhythmic grouping could result in a stronger temporal illusion. Alternatively, increased sensitivity to the elements of the groups could allow musicians to perform the duration perception task more independently from the rhythmic context resulting in a weaker temporal illusion.

In the present experiment, we used an interval discrimination task to compare participants’ processing of a temporal interval that either bordered a rhythmic group or appeared within a rhythmic group. For this purpose, we devised two tonal sequences in which the rhythm results in at least two perceptual groups [24], [25]. We chose sub-second intervals that were in the range of the highest sensitivity for temporal discrimination [26], [27]. All participants compared a target interval in a deviant sequence to an interval in a non-deviant standard sequence. This was done separately for sequences in which the target interval was *between* the perceptual groups and *within* the perceptual group. We expected a longer subjectively perceived duration of the interval *between* the groups compared to *within* a group despite the fact that the intervals were objectively identical. The subjectively perceived duration was measured by means of the point of subjective equality (PSE) in the psychometric perception curve derived from the duration discrimination task. Furthermore, we investigated the just-noticeable difference in duration that participants could perceive in the two experimental conditions. This was done by measuring the relative difference limen (DL). It is assumed that Weber’s law holds for duration perception below 1.5 s [28], although some researchers suggest a more particular relationship between duration difference perception and duration [29], [30]. Consequently, we expected that a potential duration illusion effect would also affect the just-noticeable differences resulting in higher DL for the *between*-group compared to the *within*-group condition.

## Materials and Methods

### Participants

Twenty-eight participants performed the experiment. Thirteen participants reported little or no musical training (9 females, age 23.7±4.9 y), and fifteen participants were professional musicians with graduate musical training (4 females, age 25.4±4.0 y). Participants gave written informed consent in accordance with procedures approved by the MIT committee on the use of humans as experimental subjects (COUHES) and according to the World Medical Association Helsinki Declaration as revised in October 2008. Participants were paid for their participation. All the participants had normal hearing and no history of neurological or psychiatric diseases.

### Procedure and Apparatus

Participants performed a two-alternative forced choice rhythm comparison task on a presented tone sequence. In each trial, participants were first presented with a standard rhythm sequence (probed standard rhythm, SR) and subsequently presented with a deviant rhythm sequence (comparison rhythm, CR). In the CR, the third interval was either lengthened or shortened. The participants were asked to identify whether the interval was lengthened or shortened by stating whether the fourth tone appeared “too early” or “too late”.

### Stimuli and Design

**Figure 1:**
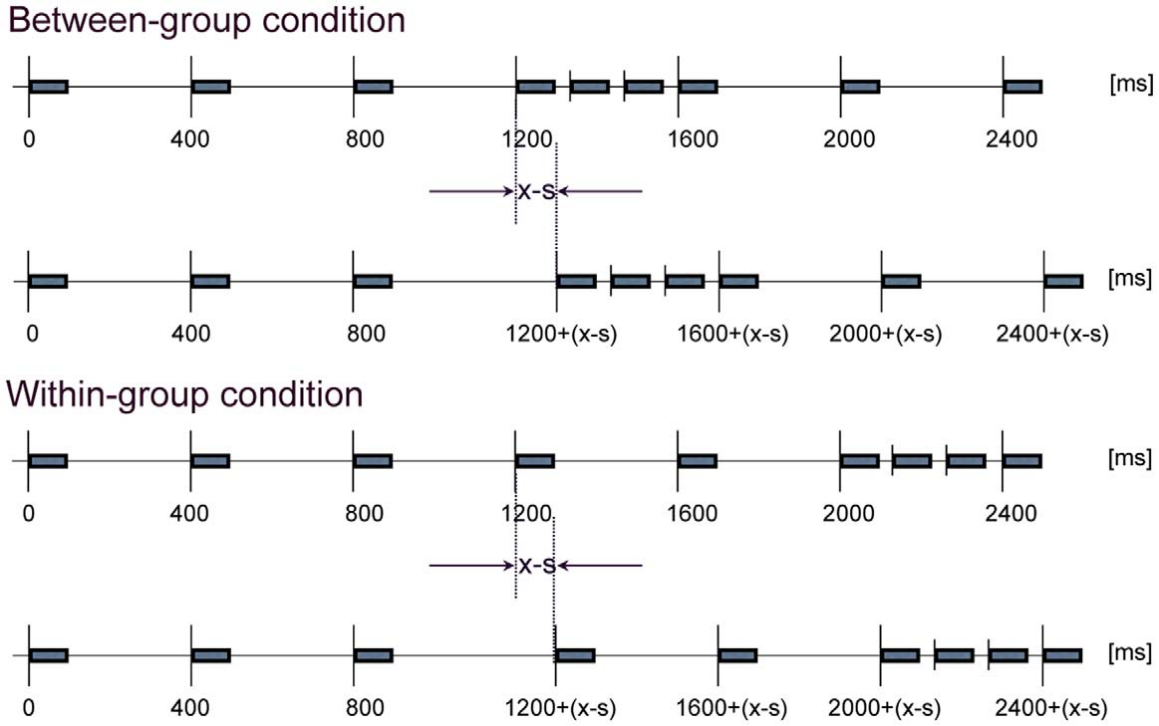
Temporal pattern of tone sequences for the *between*-group and *within*-group conditions. Top-diagrams of each condition indicate the standard rhythm (SR). Bottom-diagrams of each condition indicate the comparison rhythm (CR). x = deviant temporal interval, s = standard temporal interval.

All presented tones consisted of a fundamental frequency of 440 Hz and three harmonics with half the amplitude of the fundamental. Each tone had a duration of 80 ms and rise and fall times of 16 ms and 32 ms, respectively. The rhythmic sequences contained nine consecutive tones delimiting eight time intervals. As Figure 1 depicts, there were two experimental conditions: the *between*-group condition and the *within*-group condition. In the SR of the *between-group* condition, the first three temporal intervals (between the first four tones) were equal to T = 400 ms, the three subsequent temporal intervals were equal to T/3, and the final two intervals were equal to T. In the SR of the *within*-group condition, the first five temporal intervals were equal to T and the three subsequent temporal intervals were equal to T/3. Note that we define the duration of the time intervals as inter-onset-interval (IOI); that is, from the onset of one tone to the onset of the following tone. In the CR sequence, the third interval was either lengthened or shortened. Thus, the temporal manipulation in the CR took place either *within* a rhythmic group (the T-group) or *between* two rhythmic groups (the T-group and the T/3 group). The deviants were randomly sampled from 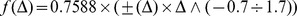 with equal number of positive and negative deviants. No deviant was repeated. The two experimental conditions were presented in pseudo-randomized order following the Kolakoski sequence [31]. The inter-stimulus interval between the two sequences, the SR and the CR, was 800 ms.

Every participant was presented with the same set of 50 trials. The standard and comparison rhythms (SR, CR) were cued by a “1” or “2” on the screen, respectively. Stimulus sequences were presented using the Psychotoolbox [32]–[34] in MATLAB (v. 2007) on a PC and presented via Sennheiser headphones (Sennheiser HD 250 Linear II) at a comfortable listening level. Psychtoolbox was also used to record the participants’ responses.

To estimate how accurately a participant could perceive the duration difference at a given standard duration 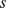, a psychometric function 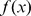, defined as the probability of giving one of the two possible answers (e.g., “fourth tone appears too late”) when presented with a deviant 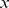, was calculated. The calculation was based on the distribution of correct answers in the task presented. We approximated the psychometric function with the equation 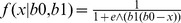, where 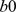 represents the point of subjective equality (PSE) and 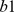 is the participant’s sensitivity to the interval duration. The difference of 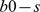 characterizes the bias toward one of the possible responses for each participant and is also referred to as the constant error (CE), where CE = PSE – point of objective equality (POE). The individual relative DL in duration perception was defined as the mean of the absolute values of deviations from the PSE that evoked the answer “too late” with 25% and 75% probability. To estimate the parameters 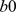 and 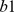 of the psychometric function for each participant, a Monte-Carlo simulation was performed. The pairs of parameters were chosen with a frequency proportional to their likelihood of occurring 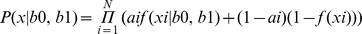 where 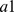 is the answer given by the participant. If the answer was “too late”, then 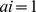. If the answer was “too early”, then 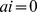. A sample of 100,000 pairs of parameters was used to calculate the relative perceptual DL of each participant as the mean over the sample and to estimate the error of the determined relative DL. We performed 2×2 repeated measures ANOVAs with a between-subjects factor (musicians/non-musicians) and a within-subjects factor (*within*/*between*) on the PSEs and relative DLs.

## Results

### Point of Subjective Equality (PSE) and Relative Difference Limen (DL)

The PSE was differentially affected by rhythmic grouping in the two groups of participants. That is, there was a significant interaction between the factors group and grouping (*F*(1,26) = 8.71, *p*<0.01, *ηρ^2^* = 0.251). Simple main effect analysis revealed that musically untrained participants showed a higher PSE *between* temporal groups (*M* = 436.1, *SE* = 8.0 ms) than *within* temporal groups (*M* = 409.7, *SE* = 8.1 ms) (*t*(12) = 3.04, *p* = 0.01, *d = 0.842*). Musicians did not show a significant difference in PSEs between experimental conditions (*p* = 0.36). Both the PSE *between* and *within* temporal groups in musicians did not significantly differ from the PSE *between* temporal groups in non-musicians (*between*: *p = 0.245*; *within*: *p = 0.605*). Furthermore, both measures did not significantly differ from the PSE *within* temporal groups in non-musicians (*between*: *p = 0.445*; *within*: *p = 0.314*). No main effect of group (*p* = 0.951) or experimental condition (*p* = 0.099) was observed.

A one-sample t-test indicated that PSEs *between* temporal groups for musically untrained participants differed significantly from the objective duration (*t*(12) = 4.20, *p* = 0.001, *d* = 1.16), whereas PSEs *within* temporal groups did not differ significantly from the objective duration (*p* = 0.319). Although musicians made duration judgments that did not differ reliably from the objective duration, it is noteworthy that they tended towards making longer duration judgments for both *between* (*p* = 0.061, *d* = 0.53) and *within* (*p* = 0.069, *d* = 0.51) temporal groups.

**Figure 2:**
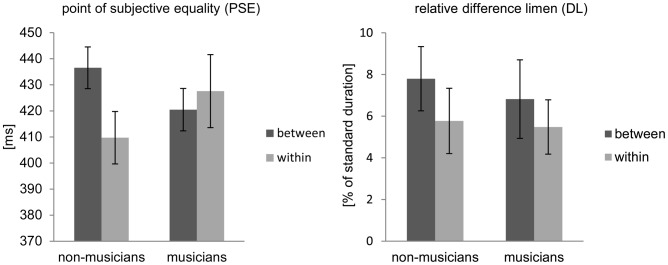
Behavioral consequences of rhythmic grouping perception. Absolute average point of subjective equality (PSE, left) and relative difference limen in percent of standard duration (DL, right). Data are plotted separately for musicians and musically untrained participants in the *within* and *between* temporal group conditions. Error bars indicate standard error. DL significantly differs between experimental condition (p<0.01). On PSE groups of participants and experimental conditions interact (p<0.01). PSE for musically untrained participants significantly differs between experimental conditions (p = 0.01).

All participants, regardless of musical training experience, perceived temporal increments in an empty interval of 400 ms significantly better when the interval was presented *within* groups (*M* = 6.2, *SE* = 1% of the standard duration) rather than *between* groups (*M* = 7.5, *SE* = 1% of the standard duration) (*F*(1,26) = 4.72, *p*<0.05, *ηρ^2^* = 0.154) (Figure 2). No interaction (*p = *0.66) or group difference (*p* = 0.71) was observed in the relative DL.

## Discussion

We asked whether auditory, rhythm-induced perceptual grouping elicits a duration illusion for a pause between tones, and how musical expertise might influence such an illusion. Participants performed a duration discrimination task on a temporal interval embedded in a rhythmic sequence of tones. We estimated the subjectively perceived duration and the just-noticeable duration difference of a target temporal interval. In musically naïve listeners, rhythmic grouping modulated perception of interval durations by an illusory lengthening of an interval presented *between* two rhythmic groups. This effect was not found in musically trained individuals. Both, musicians and non-musicians displayed increased sensitivity to duration changes when they occurred *within* a perceptual group compared to *between* rhythm-induced perceptual groups.

These results provide new insights into mechanisms of auditory rhythm perception in musically naïve participants. An illusory effect that subjectively lengthens intervals *between* perceptual groups may serve to strengthen the group boundaries. Rhythm-induced perceptual groups are perceived based on the proximity of tones. Tones that are closer together are perceived as a group. The illusory duration elongation *between* perceptual groups, thus, increases the difference in duration between that temporal interval and the next following shorter interval that is *within* a perceptual group. This illusory lengthening increases the perceptual distinction between two groups of tones, consequently magnifying the perceptual salience of a rhythmic group. This mechanism likely facilitates auditory processing when listeners need to quickly parse a stream of sound into groups of related components, e.g., syntactically related units of speech. In general, mechanisms of rhythm-induced duration illusions likely underlie earlier reported perceptual categorization in rhythm [17], [24], [35]. Thus our finding raises the possibility that, rather than being a simple misrepresentation, the duration illusion is a contributing mechanism to efficient perception of temporal structure.

For musically trained compared to untrained individuals the influence of rhythm on duration perception was fundamentally different. Their subjective duration perception was not affected by rhythm-induced perceptual grouping. Earlier studies reported that musicians display a more fine-grained perception of temporal structures in experiments that required no specific task performance [20], [23] or in specific rhythm perception tasks [20], [22]. This indicates an increased sensitivity to aspects of temporal Gestalt. Perhaps musicians were able to ignore the rhythmic grouping due to this superior sensitivity. In contrast, musically naïve participants, who are less sensitive for the temporal structure, could not evade its “time-warping” effect. Taken together, these findings could indicate that musicians are able to flexibly focus or overlook rhythmic Gestalt. They might profit from rhythm-induced perception mechanisms when needed, e.g. in language perception, and ignore it when performance of a task, such as the one applied in this experiment, requires it. In the experimental design of the current study, one cannot separate the effects of musical aptitude and musical training in relation to the absence of the duration illusion.

One interpretation of the absence of the rhythm effect on perceived duration in musicians is that they have a more accurate representation of durations. Surprisingly, however, musicians showed a trend toward over-estimation of the temporal interval in both the *between*-group and *within*-group conditions. Thus, although musicians were not susceptible to the *between*-versus-*within* group illusion, they tended to consistently over-estimate the temporal intervals. The basis of this over-estimation is unclear at present, but such an over-estimation is inconsistent with the interpretation that musicians were simply more precise in their perception of durations.

Musically naïve and trained individuals were more sensitive to temporal deviants *within* rhythmic groups than *between* rhythmic groups as evidenced by their relative DL. That is, duration differences were harder to detect at boundaries of perceptual groups than *within* a perceptual group for all participants. This findings parallel earlier findings that reported reduced gap detection abilities for temporal intervals *between* perceptual groups compared to *within* perceptual groups [9], [10], and confirm an earlier suggestion that timing *between* rhythmic groups was perceived poorly [36]. Thus, both our and prior findings indicate that there is better sensitivity for duration changes *within* a perceptual group compared to *between* groups when perceptual groups are induced by rhythm.

Musical expertise did not have an effect on sensitivity to duration changes. The relative DL for the target interval (400 ms) was 6.8% in average. This result is consistent with previous reports of perceptual DL for temporal intervals of 400 ms [26], [37]. Previous studies have generated mixed findings with respect to the influence of musical expertise on the sensitivity of individuals to duration manipulation. It has been reported that musicians outperform non-musicians in the accomplishment of auditory fusion, rhythm perception, temporal discrimination, and anisochrony perception tasks [38]–[40]. However, our findings in this study agree with the results of previous investigations that do not report an effect of musical expertise on duration discrimination tasks involving either monotonic or rhythmic sequences [26], [41]. The reasons for the heterogeneity of these results could include the various criteria that have been used to define musical expertise and the heterogeneity of the experimental paradigms that have been employed in these various studies.

The question arises whether the duration difference limen is higher in the *between*-group condition than in the *within*-group condition because of the rhythm-induced duration illusion. This notion was previously suggested in the context of a gap detection paradigm [9] and would be explained by Weber’s law [28]. However, while the effects of rhythmic grouping on PSE and DL were parallel in non-musicians, this was not the case in musicians. Thus, the different pattern of results for the two measures in the two groups argues against a simple causal relation between processes indexed by those measures. The difference in perceptual sensitivity may instead result from the different perceptual salience of the two intervals independent of associated time-warping effects. Whereas the *within*-group interval is part of the perceived group, the *between*-group interval may be interpreted as background and, thus, perceptually less salient. This difference in salience may be the reason for consistently lower perceptual threshold for duration changes *within* the perceived groups compared to *between* the perceived groups.

In summary, the present findings indicate that global rhythmic Gestalt perception affects sensitivity to duration changes with higher sensitivity to changes within a rhythmical group. Furthermore, rhythm perception amplifies perceptual group boundaries by inducing an illusory lengthening of the temporal interval *between* perceptual groups. Highly trained musicians do not display this rhythm-induced illusion effect.
